# 10 kHz SCS therapy for chronic pain, effects on opioid usage: Post hoc analysis of data from two prospective studies

**DOI:** 10.1038/s41598-019-47792-3

**Published:** 2019-08-07

**Authors:** Adnan Al-Kaisy, Jean-Pierre Van Buyten, Roy Carganillo, David Caraway, Bradford Gliner, Jeyakumar Subbaroyan, Catherine Panwar, Anand Rotte, Kasra Amirdelfan, Leonardo Kapural

**Affiliations:** 1 The Pain Management and Neuromodulation Centre, Guy’s and St. Thomas’ Hospital, London, UK; 2 Multidisciplinary Pain Centre, AZ Nikolaas, St Niklaas, Belgium; 3 Nevro Corp. 1800 Bridge Parkway, Redwood City, CA USA; 4 Panwar Health, Sydney, NSW 2036 Australia; 5 IPM Medical Group, Inc., Walnut Creek, California, USA; 6 Carolina’s Pain Institute, Winston-Salem, North Carolina USA

**Keywords:** Health care, Neurology

## Abstract

Chronic pain, including chronic low back and leg pain are prominent causes of disability worldwide. While patient management aims to reduce pain and improve daily function, prescription of opioids remains widespread despite significant adverse effects. This study pooled data from two large prospective trials on 10 kHz spinal cord stimulation (10 kHz SCS) in subjects with chronic low back pain and/or leg pain and performed post hoc analysis on changes in opioid dosage 12 months post 10 kHz SCS treatment. Patient-reported back and leg pain using the visual analog scale (VAS) and opioid dose (milligrams morphine equivalent/day, MME/day) were compared at 12 months post-10 kHz SCS therapy to baseline. Results showed that in the combined dataset, 39.3% of subjects were taking >90 MME dose of opioids at baseline compared to 23.0% at 12 months post-10 kHz SCS therapy (p = 0.007). The average dose of opioids in >90 MME group was significantly reduced by 46% following 10 kHz SCS therapy (p < 0.001), which was paralleled by significant pain relief (P < 0.001). In conclusion, current analysis demonstrates the benefits of 10 kHz SCS therapy and offers an evidence-based, non-pharmaceutical alternative to opioid therapy and/or an adjunctive therapy to facilitate opioid dose reduction whilst delivering significant pain relief. Healthcare providers involved in management of chronic non-cancer pain can include reduction or elimination of opioid use as part of treatment plan when contemplating 10 kHz SCS.

## Introduction

Chronic pain, defined as pain that remains beyond normal healing time, is a debilitating group of conditions and a prominent cause of disability worldwide^1^. While acute pain is generally nociceptive pain associated with somatosensory stimuli, chronic pain is thought to involve a shift from peripheral damage to more prominent central sensitization and central nervous system mechanisms^2^. In general, the worldwide prevalence of chronic pain in developed nations is around 20% and can be grouped into seven etiologies: primary pain that is not explained by another pain condition, cancer pain, neuropathic pain, posttraumatic and postsurgical pain, musculoskeletal pain, visceral pain and headache and orofacial pain^3–7^. Chronic low back pain (CLBP) is one of the most prevalent types of chronic pain; it is the leading global cause of disability, one of the common reasons for visits to physician’s office, correlates with work absence and is therefore associated with significant economic costs^8–13^.

Management of CLBP, like other chronic pain conditions, aims to reduce pain and improve daily function. If non-pharmacological options such as bed rest, superficial heat, cryotherapy, exercise and physiotherapy are unsuccessful, pharmacological therapies are a second-line option^8,12,14^. Of these, opioids are amongst the most commonly prescribed drugs for low back pain^15^, yet their use remains controversial^16^. Limited head-to-head evidence suggests opioids to be more effective than naproxen or placebo for relieving CLBP^17,18^, but the average effect is little more than a 10-mm reduction on a 100-mm visual analog scale (VAS), a decrease that is not considered to be clinically meaningful^19,20^. In addition, opioids have well-described short and long-term side effects such as constipation, nausea, vomiting, sedation, dizziness, respiratory depression, hormonal imbalance, changes in immune response and physical dependence; opioid overdose deaths are mainly due to acute respiratory depression caused by opioids^21–23^. Moreover, opioid use for chronic pain conditions like CLBP can cause tolerance, hyperalgesia, misuse, abuse and diversion^24–27^. Therefore, international guidelines agree that opioids need to be cautiously prescribed and should be discontinued if the benefits do not outweigh risks^28^.

In 2016, the Centers for Disease Control and Prevention (CDC) published guidelines on using opioids for management of chronic pain and capped the maximum opioid dose at 90 milligrams morphine equivalent (MME)/day as the risk of abuse and misuse rises with higher doses^29^. Even at doses ≥50 MME/day, the risk of death from opioid overdose is doubled^30^. The challenge for patients and prescribers is for patients taking ≥90 MME/day, the CDC recommends other approaches to pain management and a taper and discontinue approach for the opioid^29^. However, no definite alternatives are recommended.

Therefore, for improved quality of life, patients may desire and benefit from a reduction in opioid usage just as much as they seek relief from chronic pain through additional treatment options that enable them to achieve both goals. A minimally invasive alternative to opioid therapy is spinal cord stimulation (SCS), a type of neuromodulation typically used to treat chronic intractable neuropathic pain conditions like failed back surgery syndrome (FBSS)^31^. With SCS, the painful region is stimulated using electrical pulses and the transmission of abnormal pain signals to the brain is altered^31^. Stimulation is generally provided through electrode arrays placed into the epidural space percutaneously, or through a surgical paddle lead delivered via a laminotomy. First generation SCS devices generally deliver pulse frequencies between 40 to 60 Hz with the intent of replacing the pain sensation with a stimulation-produced sensation known as paresthesia^31,32^. However, it is challenging to produce reliable and tolerable paresthesis in the axial back region^33^. For this reason, it’s use has been largely limited to leg pain. Moreover, some patients report paresthesia as an unpleasant sensation^34^ and the perceived intensity of paresthesia can increase with body movement^35^. Another challenge of previous devices and new iterations of these devices is providing durable pain relief for back and leg pain. Multiple studies demonstrated that approximately 50% of the patients achieve ≥50% pain relief with traditional SCS and even in the patients who respond initially, therapy effectiveness was found to diminish with time^36–44^.

More recently, SCS has evolved into a variety of different modalities. One of these is 10 kHz SCS therapy that applies a 10,000 Hz waveform and provides paresthesia-free pain relief^31,45–47^. Its use has been approved for patients with chronic refractory pain of the trunk and/or limbs and is particularly suitable for CLBP.^31,45,48,49^. Two studies described previously (SENZA-EU and SENZA-RCT) showed 10 kHz SCS therapy relieves low back pain and leg pain for up to 24 months post-implant^50,51^. Compared to traditional SCS, 10 kHz SCS demonstrated superior pain relief as well as long-term improvement in quality of life in subjects with leg pain and/or CLBP^51,52^.

Despite their increasing use, to date there has been limited investigation on the role of SCS in facilitating a reduction or elimination of opioids in chronic pain management^53^. Prospective studies have shown that traditional SCS can reduce reliance on medication for management of pain. In a randomized controlled trial, North *et al*. reported that 87% (20/23) of FBSS patients in the SCS group were either stably using opioids or decreased their opioid use and only 13% (3/23) patients needed to increase their use of opioids whereas 58% (15/26) patients had a stable or decreased opioid use and 42% (11/26) patients required increased opioid use in reoperation group^37^. The results were further supported by systematic reviews on safety and efficacy of SCS for chronic pain including CLBP and FBSS, which reported stable or reduced medication usage in 65% and 53% patients treated with SCS respectively^41,54^. However, the definition of medication included opioid as well as non-opioid drugs in both studies and so far, to our knowledge, no chronic pain SCS study has assessed reduction in opioid dose as the primary endpoint and none have explored the ≥90 MME high-risk category.

This post hoc analysis of the SENZA-EU and SENZA-RCT studies examines the effect of 10 kHz SCS therapy on opioid analgesic pain management in the ≥90 MME high-risk category for subjects with leg pain and/or low back pain. This analysis will determine if 10 kHz SCS therapy may facilitate successful reduction of opioids in subjects taking the ≥90 MME doses for management of chronic pain in routine clinical practice.

## Results

### Patient demographics

In SENZA-RCT, 89 subjects were successfully included through 12 months and in SENZA-EU, 68 subjects were included through 12 months. Out of the subjects with pain relief data, 83 subjects in SENZA-RCT and 54 subjects in SENZA-EU (137 subjects in the combined population) had opioid usage data at baseline or 12-month assessment. They were included in initial analyses of demographics and clinical characteristics.

As listed in Tables 1, 60.2% subjects were female in SENZA-RCT, 53.7% in SENZA-EU and 57.7% in combined dataset. The average age of the combined population was 51.8 ± 11.7 years (mean ± standard deviation [SD]) and the mean duration since diagnosis was 11.3 ± 9.5 years. Majority of subjects reported having previous back surgery (85.4%) and were diagnosed with FBSS (81.8%). Due to minor differences in inclusion criteria between SENZA-EU and SENZA-RCT studies, such as requirement of back pain with VAS score ≥5 cm regardless of leg pain in the former study and requirement of both back and leg pain VAS scores ≥5 cm in the latter study, the baseline average back pain VAS score (mean ± standard error of the mean [SEM])was 1 cm higher than SENZA-EU study (8.4 ± 0.2 cm compared with 7.5 ± 0.1 cm), while the average leg pain score was 2 cm lower (5.1 ± 0.4 cm compared with 7.1 ± 0.2 cm). In the combined dataset, 39.5% of subjects were taking >90 MME dose of opioids prior to 10 kHz SCS treatment; 48.0% in SENZA-RCT and 27.8% in SENZA-EU.

**Table 1:** Baseline demographics and clinical characteristics in SENZA-RCT, SENZA-EU and the combined dataset.

	SENZA-RCT (N = 83)	SENZA-EU (N = 54)	Combined (N = 137)
Age, mean (SD)	52.9 (12.9)	50.0 (9.4)	51.8 (11.7)
Female, %	60.2%	53.7%	57.7%
Years since diagnosis (SD)	12.5 (10.6)	9.4 (7.4)	11.3 (9.5)
**Pain diagnoses**			
Failed back surgery syndrome	66 (79.5%)	46 (85.2%)	112 (81.8%)
Previous back surgery	86.7%	83.3%	85.4%
Back pain VAS, mean (SEM)	7.5 (0.1)	8.4 (0.2)	7.8 (0.1)
Leg pain VAS, mean (SEM)	7.1 (0.2)	5.1 (0.4)	6.3 (0.2)
Baseline opioid dose (SEM)	112.7 (10.5)	92.3 (15.6)	104.2 (9.0)
Subjects taking >90 MME opioid dose (%)	36 (48.0%)	15 (27.8%)	51 (39.5%)

### Mean opioid reduction

**Figure 1:**
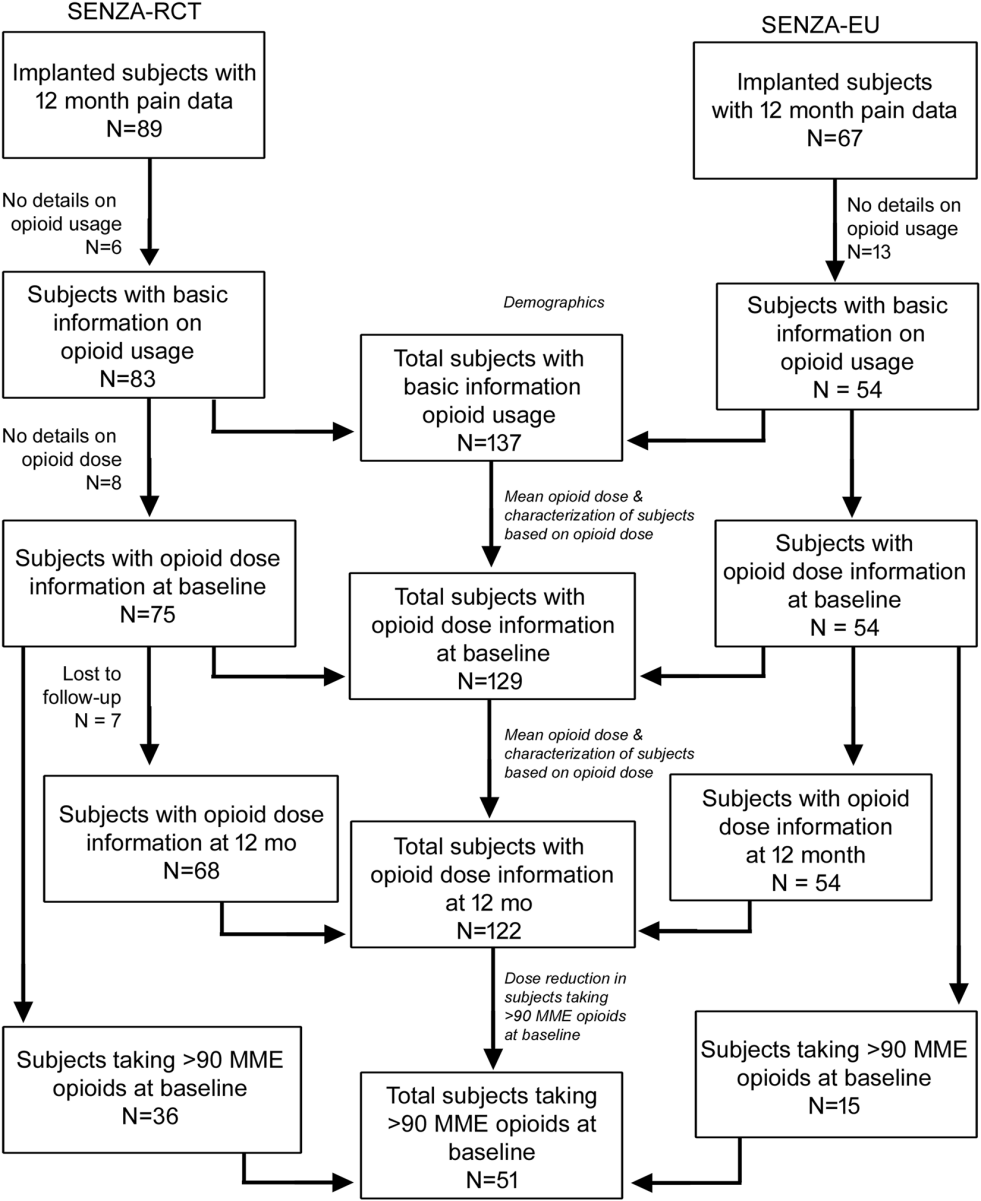
Study flow diagram showing patient datasets used for analyses.

Further analyses on mean opioid reduction, reduction in pain intensity scores and classification of subjects based on opioid dose were carried out in subjects with information on opioid dose. At baseline, dose information was available in 75 subjects in SENZA-RCT, 54 subjects in SENZA-EU and 129 subjects in the combined dataset (Fig. 1). At 12-month follow-up, dose information was available in 68 subjects in SENZA-RCT, 54 subjects in SENZA-EU and 122 subjects in the combined data set (Fig. 1).

**Figure 2:**
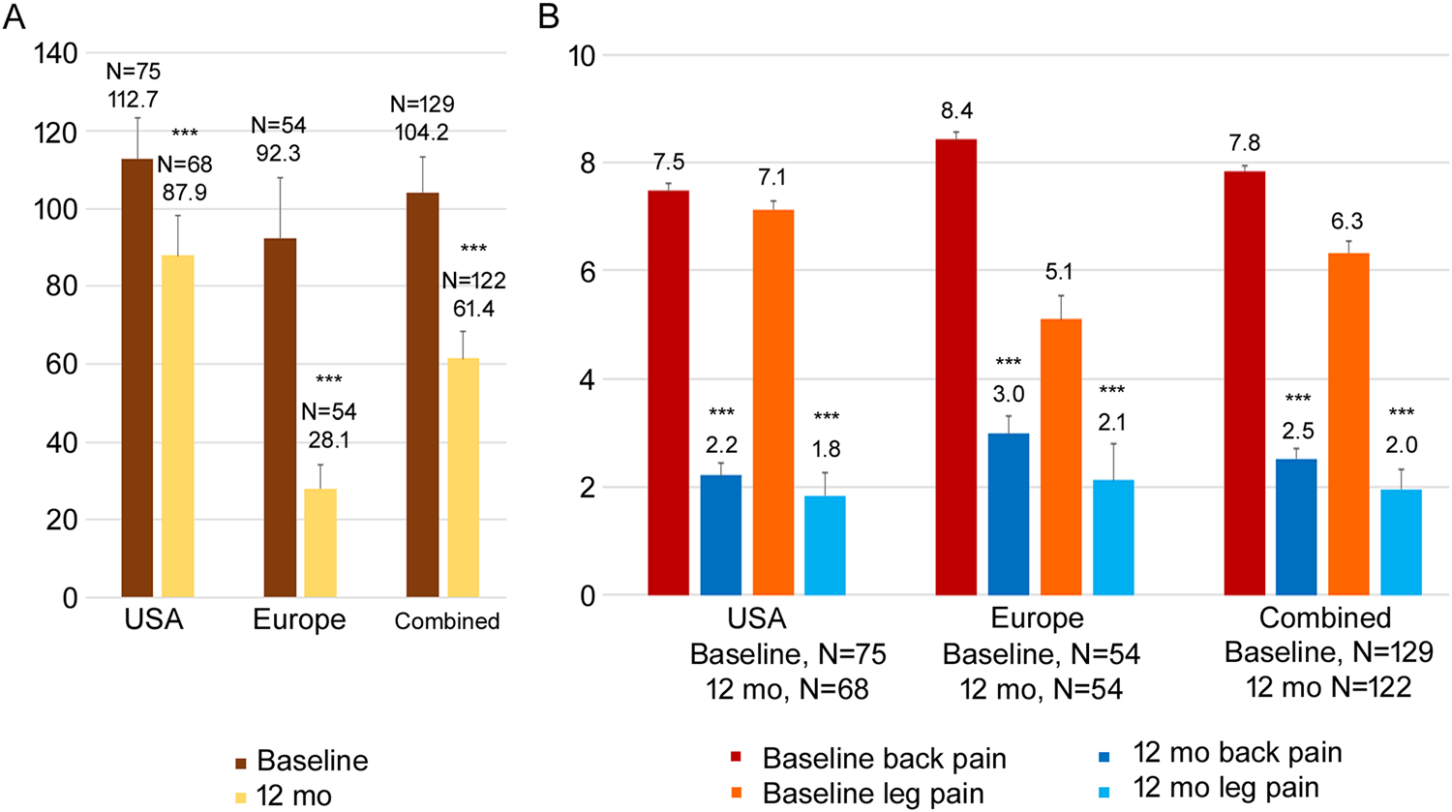
Mean opioid dose (**A**) and patient-reported back and leg pain (**B**) in SENZA-RCT, SENZA-EU and the combined dataset at baseline and 12 months following 10 kHz SCS therapy. Bars show the mean ± SEM. BL, baseline; mo, months. ***p < 0.001, paired t-test.

As illustrated in Fig. 2, though the primary endpoint of the studies was pain scores and opioid reduction was not emphasized in the study, decreases in opioid dosage were noted in the subjects following 10 kHz SCS treatment. In both studies, there was a reduction in the mean opioid dose 12-months following 10 kHz SCS therapy compared with baseline (Fig. 2A). In the combined dataset, there was a 41.0% reduction (p < 0.001) in the mean opioid dose at 12 months from 104.2 ± 9.0 MME (N = 129) to 61.4 ± 6.9 MME (N = 122). Separately, in the SENZA-EU study, the mean opioid dose at 12 months post-10 kHz SCS therapy significantly reduced from 92.3 ± 15.6 MME (N = 54) to 28.1 ± 6.0 MME (N = 54, p < 0.001). Whereas in the SENZA-RCT study, the mean opioid dose significantly decreased from 112.7 ± 10.5 MME (N = 75) to 87.9 ± 10.3 MME (N = 68) at 12 months post-10 kHz SCS therapy (p < 0.001).

### Pain response

When the studies were compared for pain reduction following 10 kHz SCS therapy, there were significant reductions in pain relief (VAS score) across both back and leg pain (Fig. 2B, p < 0.001). Overall in the combined dataset, there was a 67.9% reduction in back pain from 7.8 ± 0.1 cm (N = 129) to 2.5 ± 0.2 cm (N = 122) and 68.2% reduction in leg pain from 6.3 ± 0.2 cm (N = 129) to 2.0 ± 0.2 cm (N = 122) reported by subjects that were opioid users at 12 months post-10 kHz SCS therapy compared with baseline.

### Proportion of subjects and mean MME

**Figure 3:**
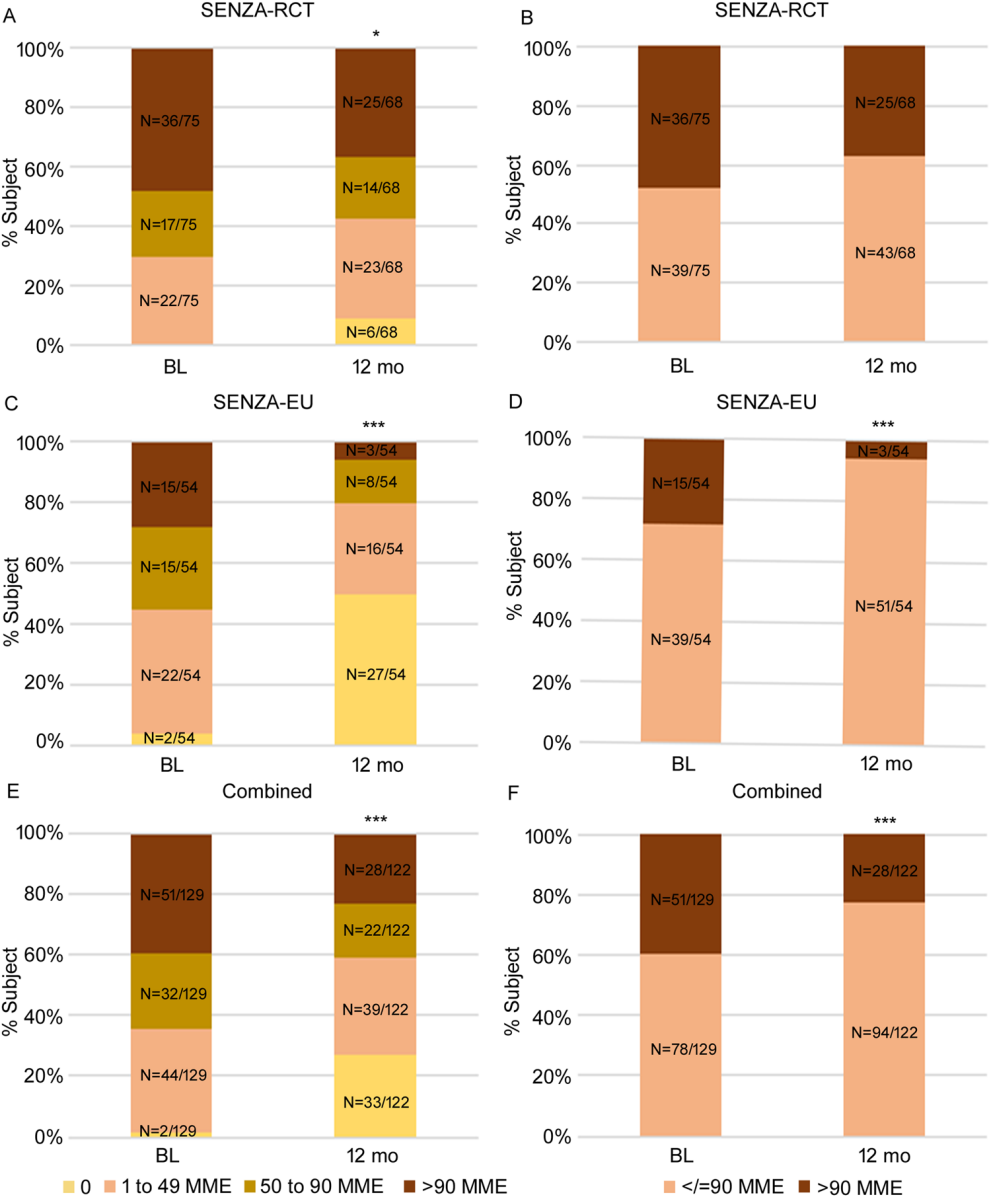
Change in opioid dose categories in SENZA-RCT (top), SENZA-EU (middle) and the combined dataset (bottom). Distribution of subjects by morphine milligram equivalent (MME) doses of opioids and distribution of high-risk (>90 MME) versus lower-risk (≤90 MME) subjects at baseline and 12 months following 10 kHz SCS therapy. BL, baseline; mo, months. ***p < 0.001, *p < 0.05 chi-square test; ^###^p < 0.001, Fischer’s exact test.

Changes in medication and opioid doses (MME) are shown in Table 2 and Fig. 3. In the combined dataset at 12 months post-10 kHz SCS therapy, 27.0% (N = 33/122) of subjects were taking no opioids, 23.0% (N = 28/122) of subjects were on >90 MME compared with only 1.6% (N = 2/129) and 39.5% (N = 51/129) at baseline, respectively (Fig. 3E, p < 0.0001). Across all doses in both SENZA-EU and SENZA-RCT, there was a significant shift towards lower MMEs following 10 kHz SCS treatment (Fig. 3C, p = 3.9 × 10^−7^ and Fig. 3A, p = 0.046 respectively).

**Table 2:** Medication changes at 12-month.

	SENZA-RCT N (%)	SENZA-EU Europe	Combined
Completely off	6 (8.8%)	27 (50.0%)	33 (27.0%)
Decreased	20 (29.4%)	12 (22.2%)	32 (26.2%)
Same	36 (52.9%)	7 (13.0%)	43 (35.2%)
Increased	6 (8.8%)	8 (14.8%)	14 (11.5%)
Total	68 (100%)	54 (100%)	122 (100%)

### Proportion of subjects ≤90 and >90 MME

Figure 3B,D,F show the proportion of subjects in doses ≤90 MME and >90 MME categories. Overall there was a significant reduction in the percentage of subjects on high-risk MME doses >90 in the combined dataset (Fig. 3F, p = 0.007).

### Changes in opioid dose in subjects in high-risk category (>90 MME)

**Figure 4:**
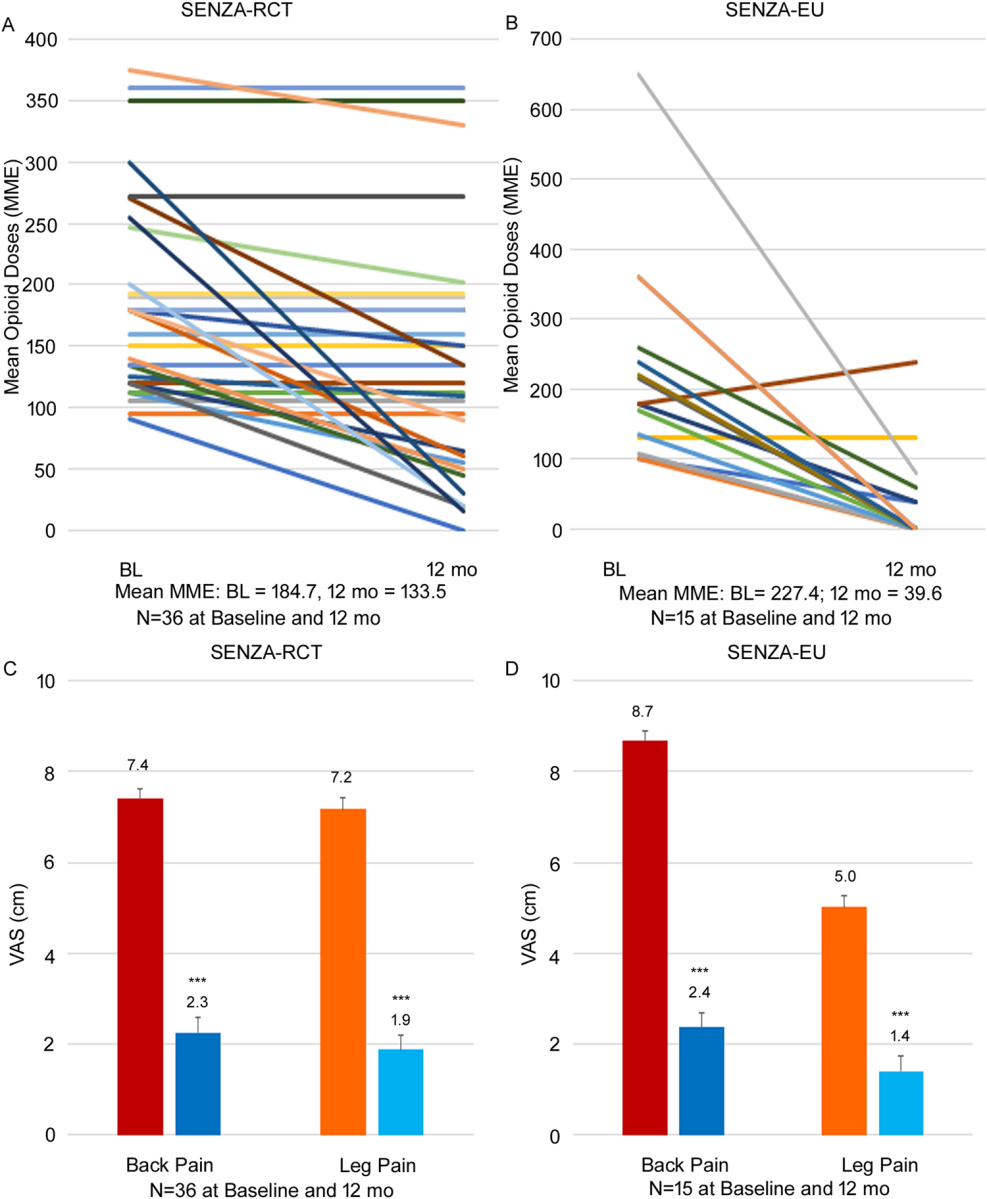
Change in opioid dose by subject in patients with a baseline MME >90 (**A**) and change in patient-reported back and leg pain (**B**) in SENZA-RCT (left) and SENZA-EU (right) at baseline and 12 months following 10 kHz SCS therapy. Bars show the mean ± SEM. BL, baseline; mo, months. ***p < 0.001, paired t-test.

Subjects taking >90 MME of opioids at baseline were then studied further for changes in opioid dose following 10 kHz SCS therapy. At baseline, 36 subjects in SENZA-RCT, 15 subjects in SENZA-EU and 51 subjects in the combined dataset were taking >90 MME of opioids for management of pain (Fig. 1). Across both studies, within the high-risk category (>90 MME) there was a trend towards a reduced opioid dose in most individual subjects 12 months post-10 kHz SCS therapy (Fig. 4A). Twelve out of 15 subjects in SENZA-EU and 11 out of 36 subjects in SENZA-RCT were able to cease or reduce their opioid dose to ≤90 MME at 12 months. Despite more subjects remaining on lower MMEs of opioids compared to baseline, significant pain reduction was reported across both studies following 10 kHz SCS therapy. As shown in Fig. 4B, following 10 kHz SCS therapy, subjects initially on >90 MME dose of opioids at baseline reported a 72.4% reduction in back pain from 8.7 ± 0.3 cm (N = 15) to 2.4 ± 0.6 cm (N = 15) and 72.0% reduction in leg pain from 5.0 ± 0.8 cm (N = 15) to 1.4 ± 0.5 cm (N = 15) in SENZA-EU and a 68.9% reduction in back pain from 7.4 ± 0.2 cm (N = 36) to 2.3 ± 0.3 cm (N = 36) and 73.6% reduction in leg pain from 7.2 ± 0.3 cm (N = 36) to 1.9 ± 0.3 cm (N = 36) in SENZA-RCT.

### Mean opioid reduction in high-risk category (>90 MME)

**Figure 5:**
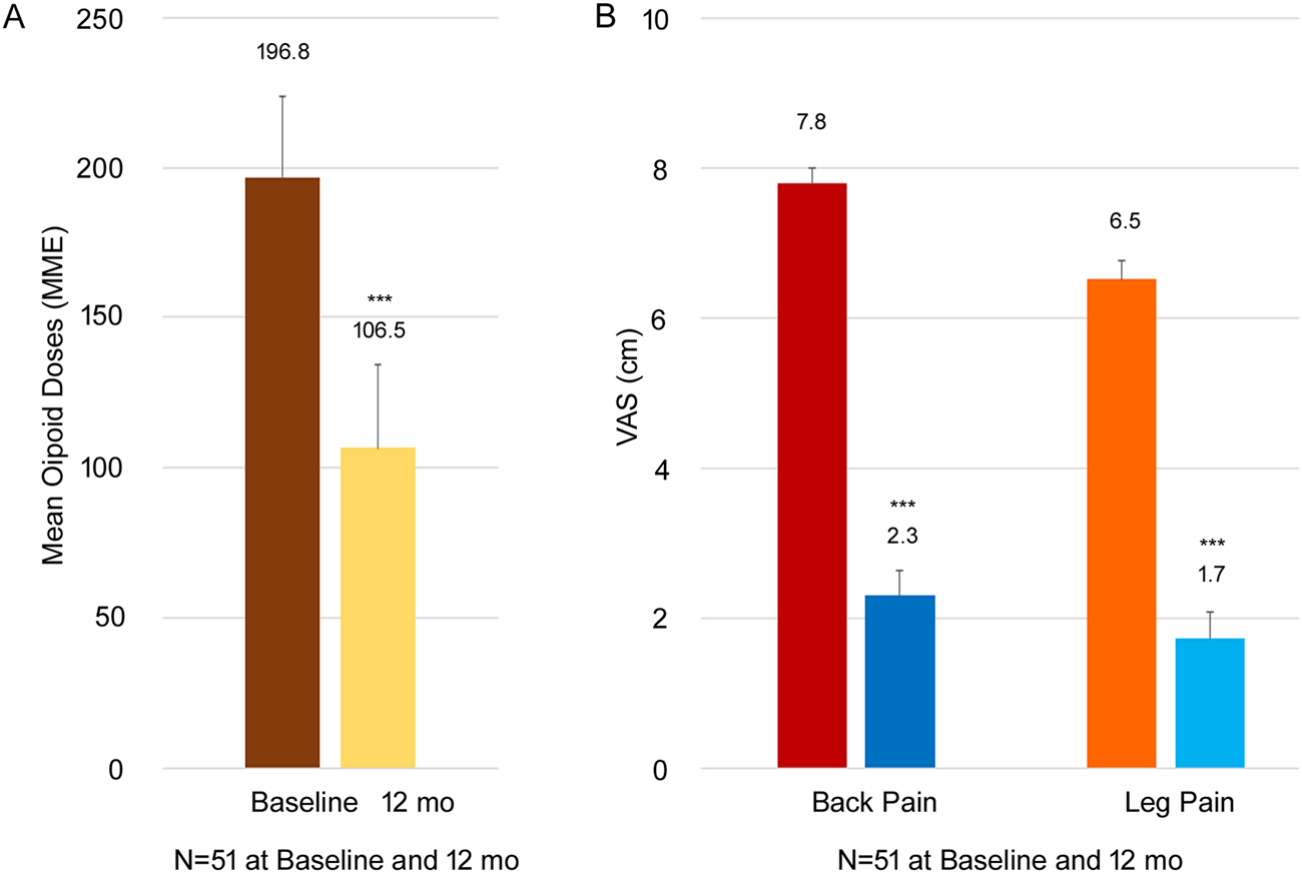
Overall reduction in mean opioid dose (mean ± SEM) in subjects with a baseline opioid dose >90 MME in the combined dataset (**A**) and change in patient-reported back and leg pain VAS scores (mean ± SEM; **B**) from baseline to 12 months following 10 kHz SCS therapy. ***p < 0.001, paired t-test.

For subjects on high doses of opioids prior to treatment (>90 MME), in the combined dataset, 12 months post-10 kHz SCS therapy resulted in a 45.9% reduction in the mean opioid dose at follow-up from 196.8 ± 14.2 to 106.5 ± 14.1 (N = 51 for both baseline and follow-up, Fig. 5A, p < 0.0001). In these subjects, significant reductions in both back and leg pain were recorded, with subjects reporting on average a 70.5% reduction in back pain and 73.8% reduction in leg pain at 12 months (N = 51 for both baseline and follow-up, Fig. 5B, both p < 0.001).

## Discussion

In North America, the opioid crisis is still at its peak^55^. Mortality rates associated with prescription opioid-related addiction and overdose have surpassed motor-vehicle accidents and patients with Human Immunodeficiency Virus, affecting people from all socio-economic classes^55^. Clear evidence is now available that demonstrates the rate of overdose of prescription opioids is directly proportional to the prescribed dose^56^. Despite efforts from regulatory bodies and clinical guidelines, opioids continue to be prescribed for patients with chronic pain conditions. In 2016, the CDC released its guideline on prescribing opioids for chronic pain and recommended avoiding or carefully justifying doses of above 90 MME^29^. Patients who do not achieve adequate pain relief and function at this dose are advised to seek alternative pain management options^29^. This recommendation received widespread criticism for its lack of guidance on suitable alternative options, leaving prescribers and patients with a dilemma regarding how to manage pain^57^. Patients are feeling left without options, and their quality of life is suffering as a consequence^58^.

Despite clear guidance on maximum doses of opioids and the rationale for high-dose opioids becoming weaker, patients continue to rely on doses > 90 MME for pain management. A recent retrospective analysis of 1,066 primary health care records of patients prescribed opioids for chronic non-cancer pain, 9.7% were receiving ≥90 MME^59^. In 2012, the CDC reported that 10% of patients on prescription opioids were receiving a high-dose (>100 MME) from one doctor, and of those who overdosed, 40% were also prescribed their pain relief from a single doctor^29^. Surveys have reported that 70% of long-term opioid users suffer from a chronic disease or disability which limits their daily function^29,60^. Assuming 10% of these patients may be on high-doses to maintain an adequate level of function and pain relief, feasible alternative options are required to reduce the risk of overdose. Clearly, patients suffering from CLBP and leg pain need evidence-based, safe and effective alternatives to long term opioid therapy.

In the current study, patients had CLBP and leg pain for an average of 11.3 ±0.8 years. They experienced significant baseline pain, despite being managed by prescription opioid therapy. Over a third (39.3%) of subjects were taking > 90 MME of opioids at baseline. 10 kHz SCS therapy was successful in reducing the mean opioid dose and patient reported pain at 12 months. Figure 2 shows following 10 kHz SCS therapy, the mean dose was reduced below 90 MME to 61.4 MME in the combined dataset. Despite a substantial reduction in the mean opioid dose, 10 kHz SCS therapy significantly improved patient-reported back and leg pain at the 12-month follow-up.

Not only was 10 kHz SCS therapy able to reduce the overall dose of opioids used by subjects with CLBP and leg pain, it reduced the proportion of subjects requiring high-risk doses >90 MME per day at the same time. Figure 3 shows in both studies fewer subjects remained on >90 MME per day, and more subjects were able to withdraw their opioid altogether following 10 kHz SCS therapy. In the combined dataset, nearly 80% of the subjects were now on dosing range associated with a reduced risk doses of opioids, with over 20% shifting from >90 MME to <90 MME at follow-up. The dose reduction was observed across individual subjects, as shown in Fig. 4, with most of them demonstrating a reduction in dose at 12 months. A reduction in pain with a reduction in opioid dose is a clinically important finding, demonstrating 10 kHz SCS therapy is not only an alternative to opioid therapy, regardless of high- or low-dose, it is able to provide more effective pain relief than baseline opioid therapy.

Interestingly, compared to SENZA-RCT, higher reductions in opioid dose and higher proportion of subjects taking lower doses of opioids at 12 months were observed in the SENZA-EU study. There may be multiple factors contributing to these observed differences. While SENZA-EU was a prospective, single-arm study designed to assess the efficacy of 10 kHz SCS system, SENZA-RCT was a randomized controlled trial designed to compare 10 kHz SCS with traditional SCS in order to support regulatory approval. Thus, the SENZA-RCT was highly focused on pain management rather than opioid reduction, whereas more emphasis was placed on weaning SENZA-EU subjects off of opioids. In SENZA-RCT, subjects were allowed to continue on stable doses of opioids but were considered as treatment failures regardless of pain scores (VAS) if they increased opioid dose. Related, there were also differences in pain management practice between study centers in SENZA-EU and SENZA-RCT. Investigators from SENZA-EU study actively encouraged the subjects to titrate and taper their opioid dose, whereas investigators from SENZA-RCT did not actively encourage opioid reduction. Lastly, chronic pain patients who are on long term opioids but still have high pain scores underscores the observation that opioids seem to have little benefit in improving chronic pain, especially as measured by VAS, yet are difficult to discontinue. Over the past decade, especially in the US, patients remained on opioids despite their dubious clinical benefit. This may have translated into clinical management changes that were not part of the goals of the RCT or EU studies.

In the past few years, several studies have documented lack of clinically relevant efficacy with the use of opioids for chronic non-cancer pain and highlighted the harmful side effects of using opioids^61–67^. The CDC guidelines published in 2016, recommended caution when increasing the opioid doses above 50 MME and avoiding opioid doses above 90 MME^29^. To prescribe safe doses of opioids and to wean the chronic pain patients off opioids, pain management specialists need durable alternatives for management of chronic pain. The current findings indicate that 10 kHz SCS can be considered as an alternative to opioids. A case-controlled retrospective study by researchers from Boston, USA showed that opioid usage was reduced or eliminated in 71% (n = 15/21) of patients treated with 10 kHz SCS for back and/or leg pain^68^. Another retrospective study by pain management specialist from Perth, Australia showed that percentage of patients not taking opioids increased nearly 2-fold in patients treated with 10 kHz SCS therapy and 70% of patients either eliminated or reduced their opioid dose at last follow-up. More importantly, average opioid dose in patients taking high dose opioids at baseline was reduced by 47% at last follow-up^69^. Current study further confirms the findings from the retrospective studies and shows that 10 kHz SCS can provide long-lasting pain relief and allow reduction or elimination of opioids even in patients who were prescribed high-risk doses. Pain physicians need to take note of the findings from the studies and encourage patients treated with 10 kHz SCS to discontinue opioids. They need to include reduction or elimination of opioid use as part of treatment plan when contemplating 10 kHz SCS. Establishing opioid reduction as part of the treatment goal even prior to the trial could help in setting up expectations and result in meaningful improvement in quality of life.

The main limitation of this study is that it involved post hoc analysis of the dataset and there was no prospectively defined statistical analysis plan (SAP) for predetermined outcomes. Although the combined dataset in the study included 129 subjects with opioid dose information, there were differences between the two studies and the number of subjects taking > 90 MME was relatively small. Additional prospective studies and real-world studies may be needed to validate the findings of this analysis. Nevertheless, current analysis shows that despite the differences between the two studies and the patient cohorts, 10 kHz SCS therapy resulted in significant reduction in opioid dosage while maintaining pain relief when the data was analysed individually and when combined.

Based on the results of this study, 10 kHz SCS therapy has the potential to assist physicians and patients in the wake of the opioid crisis, offering a non-pharmacological alternative to the suite of pain management tools for patients on high-risk doses of opioids (>90 MME).

## Methods

### SENZA-EU

As described previously, SENZA-EU was a prospective, multicentre, open-label study in 83 subjects conducted at two European centers including AZ Nikolaas Pain Centre, St Niklaas Belgium and Guy’s and St Thomas’ Pain and Neuromodulation Centre, London, United Kingdom^50^. The study protocol and informed consent forms were approved by each study site’s ethic committee (Commissie voor Medische Ethiek AZ Nikolaas, Belgium and NRES Northern & Yorkshire REC, UK respectively). Local clinical research and data protection regulations, good clinical practice guidelines (ISO 14155), and the Declaration of Helsinki were followed during the study. Selection bias in the study was minimized by the involvement of independent medical monitors, which assured high level diligence for the recruitment of subjects. Inclusion and exclusion criteria for enrollment of patients has been previously reported^50^. Briefly, patients who signed informed consent forms, with chronic back pain (defined as lumbo-sacral pain) with or without leg pain with pain intensity of ≥5.0 cm (average score over the last 30 days) on the 0 to 10 cm, visual analog scale (VAS), who were ≥18 years of age and who were candidates for SCS were included in the study. Patients were evaluated at baseline and then received a trial 10 kHz SCS (Senza® SCS system Nevro Corp., Redwood City, CA, USA) therapy for 14–30 days^50^. Patients who had at least 50% reduction in pain intensity received a permanent IPG implant and followed for 24 months with assessments at 1, 3, 6, 12, and 24 months^50^. Subjects were enrolled in the study between August 2009 and February 2011. The follow-up for the last implanted patient was completed in April 2013. The study design allowed adjustment of stimulation parameters and clinical judgement-based adjustment to pain medication dosage throughout the follow-up period^50^.

### SENZA-RCT

SENZA-RCT was a prospective, randomized, controlled trial in 198 patients across 10 centers in the U.S., designed to assess 10 kHz SCS therapy as compared with traditional low frequency SCS^51^. The study was conducted in compliance with the U.S. Code of Federal Regulations and recommendations guiding physicians in biomedical research by the 18th World Medical Assembly, Helsinki, Finland. The study protocol and informed consent forms were approved by each study site’s institutional review board (Western Institutional Review Board, Puyallup, Washington; Forsyth Medical Center Institutional Review Board, Winston-Salem, North Carolina). Selection bias in the study was addressed by the involvement of independent medical monitors for the recruitment of subjects and randomized assignment of subjects to the 10 kHz SCS group. Patients who signed informed consent forms and were candidates for SCS with chronic, intractable back and/or leg pain, refractory to conservative therapy for a minimum of 3 months with an average back pain intensity of 5 or greater out of 10 cm on the VAS, average leg pain intensity of 5 or greater out of 10 cm on the VAS, an Oswestry Disability Index (ODI) score of 41 to 80 out of 100 were randomized in a 1:1 ratio to receive stimulation with an investigational 10 kHz SCS therapy system (Senza® System; Nevro Corp., Redwood City, USA) or a commercially available SCS system (Precision Plus System; Boston Scientific, USA)^51^. Patients underwent a percutaneous trial lasting up to 14 days^51^. Those with ≥40% back pain reduction from baseline proceeded to permanent implantation and subjects with at least 50% pain reduction were considered as ‘responders’^51^. Subjects were enrolled between June 2012 and December 2012. Following randomization, subjects were implanted with 10 kHz SCS system and followed through 12 months, which concluded in February 2014. Oral analgesics were stabilized from 28 days before enrollment until activation of the implanted SCS system, excluding allowances for perioperative analgesics^51^. Adjustments were then allowed under the guidance of a study investigator as medically necessary, but the subjects who increased their opioid dose were considered as ‘non-responders’ regardless of their pain relief^51^. Assessments were performed at scheduled visits (baseline; 1, 3, 6, 9, and 12 months)^51^. Data from subjects implanted with 10 kHz SCS system was included in the current analysis.

### Outcomes

Both studies, SENZA-RCT and SENZA-EU assessed back and leg pain intensity as patient reported VAS (0 to 10 cm, no pain-to-worst pain imaginable) at baseline and 12 months. Opioid medication dose was assessed in both studies at baseline and 12 months as (milligrams morphine equivalent, MME) and the changes in opioid consumption were recorded as “eliminated”, “decreased”, “no change” or “increased”. The assessments were comparable between the studies and therefore it was possible to combine the data from the studies.

### Data collection and statistical analysis

Demographic analyses were performed in the implanted patients with data at 12 months and with basic information on opioid usage (Fig. 1). Further analyses such as mean pain intensity (VAS), mean dose of opioids (MME) and categorization of subjects based on opioid dose were performed only in data from patients with opioid dose information. Information on opioid dose was lost to follow-up at 12-month assessment in 7 subjects from SENZA-RCT study. The missing subjects were included in the calculation of mean, SEM and categorization based on opioid dose at baseline and were excluded from analyses at 12-month assessment. Two-tailed paired t-test was used for continuous variables, such as VAS and mean opioid dose and Chi-square test and Fischer’s exact test for comparison of the frequency of proportions between two groups. P-value less than or equal to 5% (p < 0.05) was considered to be statistically significant. Statistical analysis was performed using Microsoft Excel (paired t-test) and Graph Pad (Chi-square test and Fischer’s exact test) software.

## Data Availability

All data generated or analysed during this study are included in this published article.
